# The application of CRISPR gene-editing technology in influenza prevention and control

**DOI:** 10.3389/fgeed.2026.1844919

**Published:** 2026-06-25

**Authors:** Xinyi Zhang, Hangyi Shi, Jianlan Yang, Lailing Du, Xinling Zhang, Xiaoping Li

**Affiliations:** 1 Key Laboratory of Artificial Organs and Computational Medicine in Zhejiang Province, Shulan International Medical College, Zhejiang Shuren University, Hangzhou, China; 2 Gongshu District People’s Hospital of Integrated Traditional Chinese and Western Medicine, Hangzhou, Zhejiang, China

**Keywords:** crispr/cas gene editing, host susceptibility gene editing, influenza a virus, influenza B virus, lipid nanoparticle delivery, viral RNA targeting

## Abstract

Influenza A virus (IAV) and influenza B virus (IBV) remain major global public health threats because of their rapid antigenic evolution and efficient human-to-human transmission. In contrast, influenza C virus (ICV) and influenza D virus (IDV) generally exhibit narrower host ranges and milder pathogenicity, yet their potential for interspecies transmission and zoonotic spillover still warrants attention. Conventional prevention strategies, such as inactivated and live-attenuated vaccines, suffer from prolonged development timelines and diminished efficacy against rapidly evolving viral strains. However, antiviral drugs are increasingly limited by the rapid emergence of drug-resistant variants. The clustered regularly interspaced short palindromic repeats (CRISPR)/CRISPR-associated (Cas) gene-editing technology has emerged as a promising platform for influenza prevention and control owing to its programmability and precise targeting capability. In this paper, we summarize recent advances in CRISPR-based strategies for influenza prevention and control. The RNA-targeting CRISPR-associated protein 13 (Cas13) system can recognize conserved viral RNA sequences and suppress replication across influenza subtypes, whereas the DNA-targeting CRISPR-associated protein 9 (Cas9) system can edit host susceptibility genes and thereby reduce cellular permissiveness to infection. In addition, lipid nanoparticle (LNP)-based delivery systems have become important tools for improving the *in vivo* delivery and expression of CRISPR components by enhancing targeting efficiency and reducing immunogenicity. CRISPR-based diagnostics, such as Specific High-sensitivity Enzymatic Reporter unLOCKing (SHERLOCK), further expand the clinical utility of this technology by enabling rapid and sensitive detection of influenza viruses. Despite these advances, substantial challenges remain, including delivery inefficiency, off-target activity, long-term safety concerns, and the risk of viral escape. With continued technological refinement and careful translational development, CRISPR may become a versatile tool for influenza prevention, diagnosis, and therapy.

## 1 Introduction

Influenza A Virus (IAV) and Influenza B Virus (IBV) are the principal causes of seasonal influenza in humans. Infection commonly leads to fever, cough, malaise, and other systemic symptoms, and in severe cases may progress to pneumonia, acute respiratory failure, or death, particularly in the elderly, infants, and immunocompromised individuals. IAV suppresses the expression of long non-coding RNA (LRIR), thereby diminishing its inhibitory effect on transmembrane protease serine 2 (TMPRSS2) and facilitating viral replication (Chen et al., 2025). This capability enables it to cross species barriers and precipitate major influenza outbreaks (Zeller et al., 2021). Although IBV is characterized by a relatively lower mutation rate, its genome still exhibits a high mutation frequency. And it is divided into two subtypes with limited cross-immune protection between them, thereby maintaining a persistent threat to public health (Song et al., 2024).

Influenza C virus (ICV) is classified under the genus Influenza C within the Orthomyxoviridae family. It possesses a single-stranded, negative-sense RNA genome comprising seven gene segments. ICV typically causes mild upper respiratory illness, mainly in children, but recurrent infection and potential genetic exchange across species raise epidemiological concerns. Although severe complications are uncommon, recurrent infections may hinder the development of respiratory mucosa in children and present risks of cross-species genetic recombination (Lee et al., 2025). Influenza D virus (IDV), the only member of the Influenza D genus within the Orthomyxoviridae family, was first isolated from pigs. Its genome is composed of a single-stranded, negative-sense RNA with eight gene segments (Skelton and Huber, 2022). Although human infections are uncommon and generally mild, the possibility of reassortment or recombination with related viruses underscores the need for continued surveillance.

Current influenza prevention relies largely on inactivated or live-attenuated vaccines, but these approaches are constrained by lengthy development timelines and limited adaptability to rapidly evolving viral strains. Antiviral agents can reduce disease severity, yet resistance emerges readily and may compromise long-term efficacy. These limitations highlight the need for flexible, broadly adaptable strategies that can keep pace with viral evolution.

Clustered regularly interspaced short palindromic repeats (CRISPR) technology has emerged as a powerful platform for influenza prevention, diagnosis, and control. As an RNA-guided nuclease system, CRISPR enables programmable targeting of nucleic acids with high specificity and has transformed genome editing, molecular diagnostics, and functional screening. This technology facilitates highly sensitive, rapid, and accurate detection of various pathogens (Yadav et al., 2021) and offers innovative avenues for the development of broad-spectrum influenza prevention strategies. CRISPR are currently regarded as the most reliable tools for genome editing and engineering (Ishino et al., 2018), this system can achieve self-defense by identifying and cleaving viral nucleic acid sequences. Utilizing this technology, Cas proteins can be precisely edited within target cells through the use of artificially designed guide RNAs.

Importantly, CRISPR-based antiviral strategies can be divided into two complementary modes: direct targeting of viral genomes and indirect modulation of host susceptibility factors. In the former, RNA-targeting effectors such as CRISPR-associated protein 13 (Cas13) can cleave conserved viral RNA sequences and inhibit replication. In the latter, DNA-targeting systems such as CRISPR-associated protein 9 (Cas9) can edit host genes required for viral entry or replication, thereby reducing cellular permissiveness to infection. In recent years, substantial progress has been made in CRISPR-based prevention and control strategies against influenza A and B viruses, encompassing viral nucleic acid targeting and host susceptibility gene editing across various fields. These advancements enable scientists to rewrite the genetic code of nearly any organism (Davis and Yeddula, 2024). Beyond antiviral intervention, CRISPR technologies have also enabled rapid influenza diagnostics and may offer new opportunities for managing severe disease by modulating host inflammatory responses. However, several obstacles remain before these strategies can be translated into clinical practice, including efficient delivery, durable safety, off-target effects, and the possibility of viral escape. This paper summarizes the current state of CRISPR-based approaches for influenza prevention and control, with emphasis on their core mechanisms, translational delivery systems, diagnostic applications, and therapeutic prospects. It provides critical guidance for the development of next-generation, highly effective anti-influenza tools that overcome the limitations of traditional vaccines and drugs.

## 2 Core mechanisms of CRISPR system in the prevention of IAV and IBV

CRISPR systems can be broadly divided into two major classes according to the composition of their effector complexes. Class I systems require multiple Cas proteins to mediate target recognition and cleavage, whereas Class II systems rely on a single effector protein and are therefore simpler to engineer, easier to deliver, and more suitable for therapeutic development. Among the CRISPR platforms currently explored for influenza prevention and control, CRISPR-Cas13 and CRISPR-Cas9 represent the two most prominent approaches. These two systems demonstrate substantial differences in their mechanisms of action, achieving preventive effects through direct viral targeting and indirect host modification, respectively (Becirovic, 2022): Cas13 mediates direct RNA cleavage of viral genomes, whereas Cas9 primarily alters host genetic determinants of susceptibility. Together, they offer a dual strategy for influenza intervention, combining direct antiviral activity with host-directed resistance.

### 2.1 Direct antiviral activity of RNA-targeting CRISPR-Cas13 system

**FIGURE 1:**
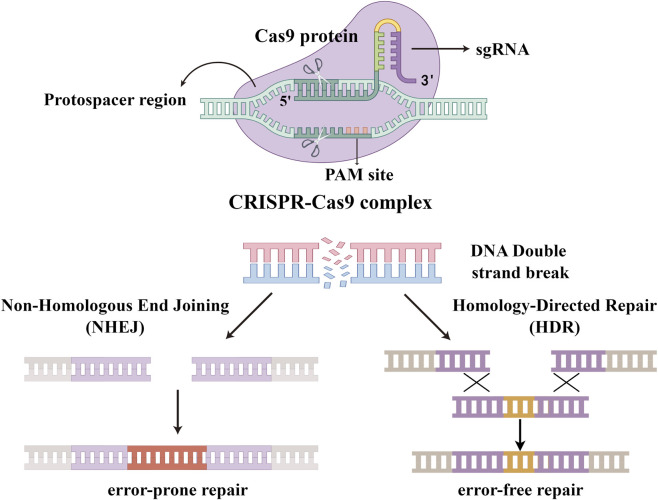
CRISPR/Cas9-Mediated Host Gene Editing Mechanism. The CRISPR/Cas9-mediated host gene editing mechanism can be divided into two pathways: non-homologous end joining (NHEJ) or homology-directed repair (HDR). Among them, NHEJ is error-prone repair, whereas HDR is error-free repair. Purple gene sequence: randomly deleted DNA sequence; Red gene sequence: randomly inserted DNA sequence; Yellow gene sequence: precisely inserted DNA sequence. This figure was drawn by Figdraw.

Cas13 is a class of effector proteins with RNAase activity, classified under the Class II CRISPR system, which includes subtypes such as Cas13a and Cas13b. Its core characteristic is the ability to specifically recognize and cleave single-stranded RNA without dependence on protospacer adjacent motif (PAM) sequences near the target, facilitating precise engineering of endogenous RNA (Shi and Wu, 2024). This PAM-independent property makes Cas13 especially suitable for targeting RNA viruses and for manipulating endogenous RNA molecules with high flexibility provides a robust platform for RNA targeting, editing, detection, and imaging, with promising biotechnological and therapeutic applications (Yang and Patel, 2024). Beyond canonical Cas13a/Cas13b, smaller and safer variants including Cas13d, Cas13X/Cas13Y, and Cas13j have been developed, differing in molecular size, cleavage specificity, and collateral activity, which directly affect their antiviral potency and *in vivo* safety profiles (Figure 1) (Freije et al., 2019; Wu et al., 2025).

The viral genomes of IAV and IBV are characterized by single-stranded negative-sense RNA, which encompasses multiple highly conserved gene sequences. These conserved sequences exhibit significant homology across various viral subtypes, rendering them optimal targets for. In experimental research, the design of CRISPR RNAs (crRNAs) that are complementary to these conserved sequences enables Cas13 to accurately identify viral RNA, thus providing an effective strategy against single-stranded RNA (ssRNA) viruses. This is achieved through the programmed cleavage of RNA that is complementary to the crRNA (Freije et al., 2019). Upon the binding of crRNA to viral RNA, the non-specific trans-cleavage activity of the Cas13 protein is activated. This activation facilitates the cleavage of a fluorescent probe, which consists of a quencher group and a reporter group, thereby permitting the detection of a fluorescence signal. Targeting strategies have advanced from single conserved sites to multiplexed crRNA cocktails against multiple essential loci such as nucleoprotein (NP), polyamide (PA), and 3′UTR, which greatly reduces the risk of viral escape caused by point mutations (Fareh et al., 2021; Man et al., 2025).

Multiple *in vitro* and *in vivo* studies have demonstrated the antiviral potential of Cas13-mediated targeting. For example, in a human alveolar chip model comprising primary human alveolar epithelial cells and pulmonary microvascular endothelial cells, crRNAs designed against conserved regions of the IAV H_3_N_2_ genome significantly reduced viral replication in the alveolar compartment. This intervention was also associated with attenuated inflammatory signaling, as reflected by reduced cytokine production and decreased immune cell recruitment. These findings suggest that Cas13-based targeting can suppress viral propagation while simultaneously limiting host inflammatory injury. Subtype-specific comparisons indicate that Cas13d exhibits the highest antiviral efficacy, followed by Cas13X/Cas13Y, with Cas13a showing relatively stronger collateral activity and cytotoxicity (Yang and Patel, 2024; Freije et al., 2019). LNP-formulated Cas13d crRNA has achieved significant viral load reduction and improved survival in lethal IAV mouse models, with minimal pulmonary toxicity (Wu et al., 2026).

However, the trans-cleavage activity of Cas13 also introduces safety concerns. Excessive or prolonged Cas13 activation may lead to unintended degradation of host RNAs, potentially causing cytotoxicity. To improve therapeutic specificity, researchers have optimized crRNA design, adjusted Cas13 expression levels, and employed human organ-on-chip platforms to better evaluate efficacy and safety in physiologically relevant settings (Man et al., 2025). These strategies provide an important foundation for future clinical translation of Cas13-based antiviral therapy. Unlike DNA-targeting Cas9, Cas13 operates exclusively at the RNA level, avoiding permanent genomic alterations or chromosomal damage, which further enhances its safety profile (Shi and Wu, 2024; Chew, 2018).

Collectively, RNA-targeting CRISPR-Cas13 systems are uniquely suited for influenza intervention, owing to their inherent compatibility with RNA viruses, high safety profile, broad-spectrum potency, and capacity for rapid adaptation to viral evolution. Unlike DNA-targeting Cas9, Cas13 directly recognizes and cleaves conserved viral RNA without requiring PAM sequences, enabling flexible targeting across diverse IAV and IBV subtypes (Freije et al., 2019). Operating exclusively at the RNA level, Cas13 avoids permanent genomic alterations, off-target DNA cleavage, or chromosomal aberrations, thus minimizing safety risk (Shi and Wu, 2024). Furthermore, its programmability allows rapid redesign of crRNAs to counter newly emerged strains, while multiplex targeting of multiple conserved regions substantially reduces the likelihood of viral escape (Chew, 2018). Coupled with its utility in SHERLOCK-based diagnostics and its compatibility with respiratory delivery via LNPs, Cas13 provides an integrated platform for influenza prevention, diagnosis, and therapy, making it a superior choice for influenza control compared with conventional vaccines, antiviral drugs, or DNA-editing strategies (Freije et al., 2019; Man et al., 2025; Abudayyeh et al., 2016).

### 2.2 Host-directed antiviral strategy based on CRISPR-Cas9

In contrast to Cas13, which directly targets viral RNA, CRISPR-Cas9 exerts antiviral effects by modifying host genes that facilitate influenza infection. Cas9 is a DNA endonuclease that recognizes target sequences in the presence of a PAM and introduces double-strand breaks. The resulting DNA damage is repaired primarily by non-homologous end joining or homologous recombination, enabling gene knockout, sequence replacement, or other forms of genome modification (Zheng et al., 2023). Cells repair DNA double-strand breaks through two primary mechanisms: non-homologous end joining (NHEJ) and homologous recombination (HR). NHEJ is more likely to introduce insertional or deletional mutations in target genes, thereby facilitating gene knockout, whereas HR allows for precise gene insertion or replacement.

Influenza virus infection depends not only on viral proteins but also on host factors that mediate entry, intracellular trafficking, transcription, and replication. This dependence creates an opportunity for host-directed intervention. By disrupting genes that encode critical susceptibility factors, CRISPR-Cas9 can reduce the permissiveness of host cells to influenza infection and establish a more durable antiviral barrier.

**FIGURE 2:**
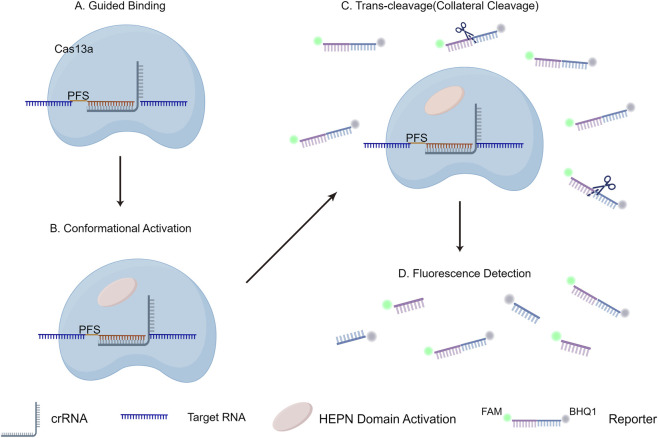
Schematic overview of the Cas13a-mediated RNA detection mechanism. This figure was drawn by Figdraw. **(A)** Guided binding: The Cas13a-crRNA complex specifically recognizes and binds to the target RNA containing the protospacer flanking site (PFS). **(B)** Conformational activation: Perfect base pairing between the crRNA and target RNA triggers a conformational change in Cas13a, leading to the activation of its HEPN nuclease domains. **(C)** Trans-cleavage (collateral cleavage): Activated Cas13a exhibits non-specific RNase activity, indiscriminately cleaving nearby single-stranded RNAs, including the fluorescent reporter probes. **(D)** Fluorescence detection: Cleavage of the reporter probes releases the fluorophore from the quencher, producing a measurable fluorescent signal that reports the presence of the target RNA.

Previous studies have identified multiple host genes that support influenza virus replication. CRISPR-Cas9-based loss-of-function screening has been used to uncover these host determinants and to validate their roles in the viral life cycle. CRISPR system variants follow a similar process, which can be delineated into three stages: adaptation, expression, and interference (Shi et al., 2021). The study identified that the transcription protein encoded by the SLC351 gene in host cells is a critical target for influenza viruses, which exploit this protein to invade host cells. The CRISPR/Cas9 system can be employed to identify key host factors involved in pathogen replication within cells (Tian et al., 2025), and the application of gene knockout techniques to target susceptible genes has proven effective in diminishing the susceptibility of host cells to the influenza virus (Figure 2).

That said, host-directed editing also raises important safety considerations. Many host susceptibility factors are involved in normal physiological processes, and their disruption may lead to unwanted biological consequences. Thus, identifying host targets that are both functionally important for infection and dispensable for normal cellular function remains a critical challenge.

### 2.3 Extended applications of CRISPR in influenza diagnosis and severe disease management

The Cas13-associated collateral cleavage activity has been harnessed for rapid influenza diagnosis, culminating in the development of Specific High-Sensitivity Enzymatic Reporter UnLOCKing (SHERLOCK) technology (Kostyusheva et al., 2022). This approach exploits the trans-cleavage activity of Cas13; upon recognition and cleavage of target viral RNA, activated Cas13 cleaves fluorescently labeled reporter RNA in the reaction system, enabling viral detection through fluorescence signal readout. Compared with conventional RT-PCR, SHERLOCK does not require complex thermal cycling instrumentation, thereby reducing detection time from 2 to 3 h to less than 30 min. It achieves a detection limit of 10 copies/μL and can distinguish among IAV subtypes, such as H_1_N_1_ and H_3_N_2_, as well as IBV. In clinical validation using nasopharyngeal swab samples from patients with influenza, this technology demonstrated a sensitivity of 92% and a specificity of 98% (Liu et al., 2025), highlighting its potential for rapid screening in primary care settings and during outbreak responses.

CRISPR technology may also have therapeutic potential in severe influenza, particularly in cases complicated by hyperinflammation and acute respiratory distress syndrome (ARDS). Severe influenza can trigger excessive cytokine production, including IL-6 and IL-1β, leading to pulmonary injury and impaired gas exchange. CRISPR-mediated editing of host inflammatory pathways, such as the IL-6 receptor axis, may therefore help attenuate pathogenic immune activation and improve clinical outcomes. In a severe IAV mouse model, delivery of IL-6R-targeting CRISPR components via lipid nanoparticles significantly reduced pulmonary cytokine levels, alleviated lung pathology, and improved survival. In a severe IAV-induced mouse model, the IL-6R gene-editing system delivered via LNPs reduced pulmonary inflammatory cytokine levels by more than 50%, significantly improved the pathological injury score of lung tissue, and improved survival from 30% to 75% (Huang et al., 2024). In addition, CRISPR-based enhancement of antimicrobial defense pathways may help reduce the risk of secondary bacterial infection, a major contributor to influenza-associated morbidity and mortality.

## 3 Optimization of CRISPR technology delivery systems

Efficient *in vivo* delivery remains one of the major barriers to the clinical translation of CRISPR-based influenza interventions. Both Cas proteins and guide RNAs are large biomolecules that are vulnerable to degradation by nucleases and proteases, and they also face substantial obstacles in crossing cellular membranes and reaching the appropriate intracellular compartments.

These challenges are uniquely pronounced in the lung, where layered biological barriers, including a dense, negatively charged mucus layer (5–10 μm thick), rapid mucociliary clearance, and tightly sealed epithelial monolayers, complicate targeted delivery to respiratory epithelial cells, the primary site of influenza infection and replication (Fareh et al., 2021). IAV and IBV viruses initiate infection by binding sialic acid residues on the apical surface of bronchial and alveolar epithelial cells, followed by endocytosis and reliance on host factors such as TMPRSS2 and SLC35A1 for uncoating and genome replication (de Lima et al., 2025; Han et al., 2018). This intimate virus–host interaction makes it essential to deliver CRISPR components directly to these epithelial cells to block infection at the source.

Conventional vehicles are frequently trapped in the mucus layer, cleared by ciliary movement within minutes, or non-specifically taken up by alveolar macrophages, resulting in insufficient target-site accumulation and reduced editing efficacy (Sun et al., 2026). Among current delivery approaches, lipid nanoparticle (LNP)-based platforms have emerged as the most promising for respiratory delivery, while viral and other non-viral systems persistently face limitations in safety, packaging capacity, or targeting precision.

### 3.1 Lipid nanoparticle delivery system

LNPs are nanoscale entities formed through the self-assembly of lipid constituents, capable of encapsulating negatively charged CRISPR components via electrostatic interactions (Wu et al., 2026), thereby ensuring their protection and targeted delivery. These nanoparticles typically comprise four principal lipid components: ionizable cationic lipids, polyethylene glycol (PEG) lipids, phosphatidylserine, and cholesterol. LNPs predominantly internalize into cells through the endocytic pathway. The distinct components of the LNP system confer unique functional attributes that collectively facilitate the encapsulation, transport, and delivery of payloads. The ionizable lipids play a crucial role in enhancing the encapsulation and delivery of nucleic acids by LNPs, acting as the core component responsive to pH variations. In a neutral environment, ionizable lipids remain electrically neutral, contributing to the stability of the LNPs. Upon cellular entry, the acidic milieu of the endosome protonates the ionizable lipids, imparting a positive charge. This charge interacts with the negatively charged phospholipids of the endosomal membrane, disrupting its integrity and promoting the release of LNP-encapsulated substances into the cytoplasm. This process, known as “endosomal escape,” is a pivotal step in determining the delivery efficiency of LNPs (Kiaie et al., 2022).

To address the mucus barrier, recent LNP designs favor charge-neutral or weakly anionic surfaces at physiological pH, minimizing electrostatic interactions with negatively charged mucins and improving penetration through airway mucus (Kumari et al., 2022). PEGylated lipids form a hydrophilic protective layer on the LNP surface, which reduces aggregation and clearance *in vivo*, thereby extending the circulation half-life. Additionally, PEGylated lipids decrease the immunogenicity of LNPs, thus attenuating the host immune response. Phospholipids are predominantly situated in the outer lipid layer of LNPs, where they enhance structural stability and delivery efficiency, while also improving membrane fluidity and fusion capacity. Cholesterol is integrated into the LNP formulation to enhance particle stability by occupying interphospholipid spaces. Furthermore, cholesterol facilitates membrane fusion during cellular uptake by enhancing the activity of positively charged lipids and destabilizes the cell membrane during the fusion process (Kazemian et al., 2022; Mülle et al., 2024).

For epithelial targeting, surface modification with sialic acid-mimetic peptides or influenza hemagglutinin (HA) aptamers enables LNPs to specifically bind to sialic acid receptors on bronchial and alveolar epithelial cells, increasing cellular uptake and specificity for influenza-susceptible cells (Chang et al., 2021). In studies focused on the prevention of IAV and IBV, the LNPs delivery system offers a significant advantage due to its capacity for targeted delivery to the respiratory tract. Researchers have investigated the use of LNPs to deliver CRISPR-Cas9 as a dry powder to the lungs, specifically targeting respiratory epithelial cells, which are the primary sites of influenza virus infection. This approach involves transporting LNPs encapsulating CRISPR components, thereby increasing the likelihood of achieving high efficacy while minimizing adverse effects (Carneiro et al., 2023). Animal experiments have demonstrated that the LNP-Cas13/crRNA complex, when delivered in this manner, effectively penetrates the respiratory mucosal barrier in mice, is absorbed by pulmonary epithelial cells, and substantially reduces viral loads of IAV and IBV in the lungs without causing significant pulmonary inflammatory damage (Haley et al., 2025). Additionally, the targeting capability of LNPs can be further enhanced by modifying the ligand, thereby reducing off-target effects on non-target organs.

### 3.2 Viral delivery system

The viral delivery system leverages the inherent infectivity of viruses to facilitate the delivery of CRISPR components into target cells. Adeno-associated virus (AAV), a defective single-stranded DNA virus composed of capsid proteins and single-stranded DNA (Du et al., 2023), is distinguished by its high transduction efficiency, low immunogenicity, and tissue specificity, making it the most extensively utilized and promising vector in gene therapy. Researchers can incorporate the Cas gene and guide RNA (gRNA) encoding sequence into the genome of an AAV vector, which can then be administered via the nasal cavity or trachea. This approach allows the AAV to infect respiratory epithelial cells, thereby inducing the expression of CRISPR components to facilitate gene editing.

For influenza applications, the AAV6 serotype exhibits strong tropism for human bronchial and alveolar epithelial cells, enabling efficient delivery of CRISPR components to the primary site of influenza replication (Chen et al., 2022). Researchers can incorporate the Cas gene and guide RNA (gRNA) encoding sequence into the genome of an AAV vector, which can then be administered via the nasal cavity or trachea. This approach allows the AAV to infect respiratory epithelial cells, thereby inducing the expression of CRISPR components to facilitate gene editing.

Nonetheless, viral delivery systems are associated with several limitations. First, packaging constraints limit the size of CRISPR components that can be delivered, making large Cas9 variants difficult to package while accommodating multiple gRNAs (Karimi et al., 2026). Second, pre-existing anti-AAV antibodies in a significant portion of the human population reduce transduction efficiency and limit repeated administration (Chandra et al., 2023). Third, random integration of AAV into the host genome raises potential genotoxic risks in lung epithelial cells (Chen et al., 2022).

For example, AAV vectors face challenges in concurrently delivering the large-molecular-weight Cas gene along with multiple gRNA coding sequences. Their intrinsic immunogenicity can provoke immune responses, resulting in vector clearance and diminished editing efficacy. Moreover, the random integration of viral vectors can cause insertional mutations within the host genome, thereby presenting potential safety concerns (Khoshandam et al., 2024). Consequently, this approach is predominantly utilized in cell and animal models for basic research, with its application in clinical influenza prevention studies being relatively limited.

### 3.3 Other delivery systems

In addition to LNP and viral vectors, researchers have developed a variety of innovative CRISPR/Cas9 delivery systems. These include solid delivery methods using physical techniques, as well as polymer nanoparticles and inorganic nanocarriers (Du et al., 2023).

Polymer nanocarriers, such as chitosan and polyethyleneimine (PEI)-based nanoparticles, can enhance mucus penetration and deliver CRISPR components to respiratory epithelial cells via charge shielding and endosomal escape mechanisms (Sato, 2025). Among the various physical delivery systems, electroporation, microinjection, and hydrodynamic tail vein injection (HTVI) are prominent methods for delivering CRISPR components via physical mechanisms. These techniques are compatible with all CRISPR components and are predominantly employed in *in vitro* cell experiments. Although HTVI can target hepatic components (Sun et al., 2024), its limited targeting specificity and the significant tissue damage it causes *in vivo* pose challenges for clinical translation in influenza-related applications. Conversely, the polymer nanocarrier system, utilizing polyethyleneimine (PEI), Polyamidoamine (PAMAM), and chitosan as core components, forms polymers through charge neutralization to encapsulate CRISPR components such as Cas9/gRNA and Cas13/crRNA. These encapsulated components are internalized into cells via endocytosis and subsequently achieve endosomal escape through the proton sponge effect, thereby safeguarding nucleic acid stability (Wu et al., 2026) (Ma et al., 2022). While primarily employed *in vitro*, small quantities can be directed to the lungs, offering valuable insights for influenza pulmonary research.

Inorganic nanocarriers, including gold and silica nanoparticles, can be surface-modified to target influenza-infected epithelial cells, although their *in vivo* toxicity and biodegradability remain concerns (Siafaka et al., 2024). These systems predominantly employ gold nanoparticles and silica nanocarriers, which are surface-modified to target specific cells. Following endocytosis, these systems release CRISPR components (Rakotondrabe et al., 2023) that are compatible with all necessary elements. Presently, they are currently limited to *in vitro* cell experiments, and their performance still needs further optimization. is required (Table 1).

**TABLE 1:** CRISPR/Cas delivery systems: carriers, mechanisms, and targets.

Delivery system type	Core carrier/Technology	Core delivery mechanism	Target site	Adapt CRISPR components	References
Non-viral delivery systems	Lipid nanoparticles (LNP)	Electrostatic interaction encapsulates components such as Cas protein/crRNA/gRNA; Endocytosis into target cells; Acidic endosomal environment triggers protonation of ionizable lipids, disrupting the endosomal membrane to achieve “endosomal escape”; Release of components into the cytoplasm/nucleus for functional effects	Respiratory epithelial cells, lung tissue	Cas9/gRNA Cas13/crRNA	Wu et al. (2026); Kiaie et al. (2022)
Physical delivery system	Electroporation/Microinjection/Hydrodynamic Tail Vein Injection (HTVI)	Electroporation: Penetration of the cell membrane by an electric field to allow components to enter the cell; Microinjection: Direct injection into the cell; HTVI: High-velocity intravascular infusion (HTVI) of components to accelerate their penetration through the vascular endothelium into tissues	*In vitro* cells, liver (HTVI)	All CRISPR components (*in vitro* preferred)	Sun et al. (2024)
Polymer nanocarrier systems	Polyethyleneimine (PEI)/Polyamine Amine (PAMAM)/Chitosan	Polymers and nucleic acids form polymers through charge neutralization; Endocytosis into cells; Proton sponge effect of polymers facilitates endosomal escape	*In vitro* cells, lungs (small amount *in vivo*)	Cas9/gRNA Cas13/crRNA	Wu et al. (2026), Ma et al. (2022)
Inorganic nanocarrier systems	Gold Nanoparticles/Silica Nanocarriers	Surface modification of aptamers/targeting molecules for cell recognition; Endocytosis into cells; Release of components under acidic conditions	*in vitro* cell	All CRISPR components (primarily *in vitro*)	Rakotondrabe et al. (2023)

## 4 Discussion

Characterized by programmability and precise targeting, CRISPR gene-editing technology provides a novel approach for the prevention and control of influenza A and B viruses (IAV and IBV), and can effectively overcome the drawbacks of traditional antiviral strategies including rapid viral variation, cross-species transmission and widespread drug resistance.

Mechanistically speaking, Cas13 can programmatically clew RNA and crRNA complementarity without relying on PAM sequences, and can specifically recognize and degrade the genomic RNA and transmrna of RNA viruses such as COVID-19 and influenza, achieving a broad-spectrum anti-RNA virus effect (Freije et al., 2019); Cas9 can eliminate the genomes or proviruses of DNA/retroviruses such as HBV/HIV, but it has limitations such as delivery constraints (large size), DNA off-target effects, the risk of chromosomal damage, and the restriction of being only applicable to DNA viruses (Bartosh et al., 2023).

Notably, RNA-targeting CRISPR systems are particularly well suited for influenza therapy because influenza viruses are single-stranded RNA viruses that replicate exclusively in the cytoplasm without DNA intermediates. This makes RNA-directed cleavage highly efficient and specific, while avoiding permanent genomic alterations associated with DNA-editing systems. The transient, reversible action of RNA targeting also improves safety, and the programmability of Cas13 allows rapid adaptation to evolving influenza strains, further supporting its translational value for influenza intervention.

In terms of delivery compatibility, subtypes such as Cas13d, Cas13X/Y have compact molecular structures, which can be easily adapted to AAV single-vector packaging and also support multiple delivery methods including mRNA-LNP. They exhibit high delivery efficiency both *in vivo* and *in vitro*, and have stronger adaptability to various vectors (Freije et al., 2019); in contrast, the SpCas9-encoding fragment is relatively large, exceeding the conventional packaging capacity of AAV. Therefore, it can only rely on small SaCas9 variants, split vectors, or LNP-RNP for transient delivery. The design of the vector and *in vivo* delivery are more restricted (Schmelas and Grimm, 2018).

In terms of safety, Cas13 only functions at the RNA level and does not cause breaks in the host DNA chain. There is no risk of permanent damage to the genome or chromosomal aberrations (Freije et al., 2019); the Cas9 editing process is prone to causing DNA double-strand breaks, and there are potential risks of off-target effects on the genome as well as chromosomal deletion and rearrangement (Reginato et al., 2024). Moreover, the proportion of people pre-existing anti-Cas9 antibodies is relatively high, and intravenous administration can easily trigger inflammatory immune responses (Chew, 2018).

When the virus escapes, Cas13 can freely select the ultra-conserved regions of the viral genes to design crRNAs. It can tolerate single nucleotide mismatches. Combined with a cocktail of multiple crRNAs, this approach can significantly reduce the probability of RNA virus mutation and escape (Fareh et al., 2021); the Cas9 system is prone to generating escape strains due to mutations in the viral PAM site or the target DNA sequence. Coupled with the latent integration characteristic of DNA viruses, it is even more likely to experience viral rebound after treatment (Zhang et al., 2025).

In the transformation potential dimension, Cas13 possesses the integrated advantages of rapid pathogen detection and antiviral treatment (Freije et al., 2019), adapted for the prevention and control of new and emerging RNA viruses, the compact molecular structure is more conducive to the modification and implementation of clinical carriers (Chung et al., 2024); Cas9, thanks to its unique ability to eliminate latent DNA viruses, has advanced to the clinical research stage for HIV infection and is the core tool for functional cure of chronic DNA virus infections (Zhang et al., 2025). Recent studies have also tended to adopt the Cas13 combined with Cas9 strategy, which can jointly inhibit free RNA viruses and clear latent reservoirs of DNA viruses, providing a new direction for the prevention and control of viral infections (Fareh et al., 2021; Baddeley and Isalan, 2021). The combined use of these two systems provides a diverse set of technical strategies for influenza prevention and control.

Optimizing delivery systems is the core link to promote the clinical transformation of CRISPR technology, among which lipid nanoparticle (LNP) delivery platforms possess prominent application advantages. Relying on ionizable lipids with pH responsiveness, PEGylated lipids with stable properties and cholesterol for membrane fusion, LNPs can efficiently encapsulate and deliver CRISPR editing components. Animal experiments have verified that this delivery system can effectively penetrate respiratory mucosal barriers, reduce viral load and maintain good biosafety, showing great potential for respiratory targeted delivery. By contrast, although adeno-associated viral (AAV) vectors feature high transduction efficiency, they are limited by insufficient packaging capacity, strong immunogenicity and potential risk of insertional mutation,and are mostly confined to basic research. Other emerging delivery strategies such as polymer nanoparticles and physical delivery methods still need further optimization to improve practical applicability.

At present, the clinical popularization of CRISPR technology in influenza prevention and control still faces multiple obstacles, mainly focusing on insufficient delivery efficiency and inaccurate targeting. As the mainstream delivery vehicle, LNPs are difficult to stably cross respiratory mucosal barriers and accurately locate respiratory epithelial cells. Nanoparticles are easily trapped by mucus or eliminated through non-specific uptake, leading to insufficient enrichment of editing components at lesion sites and declined gene editing efficiency (Leong and Ge, 2022). Meanwhile, LNPs lack cell-type targeting specificity and are prone to being internalized by respiratory immune cells, further weakening antiviral effects (Healy et al., 2025).

In terms of safety, Cas proteins may exhibit off-target activity. Prolonged administration may increase the risk of nonspecific mutations in the host genome, potentially leading to abnormal cell proliferation and downstream pathological consequences such as tumorigenesis (Qiu et al., 2021). Continuous expression of exogenous Cas proteins can also trigger host immune rejection, and the long-term cumulative toxicity of LNP delivery systems remains to be systematically evaluated. The high mutability of influenza viruses also creates a risk of viral escape; even when conserved sequences are targeted, viruses may evade Cas9-mediated cleavage through point mutations that disrupt recognition (Hossain et al., 2021). Although multi-target editing can alleviate this problem, it also increases the difficulty in component design and delivery preparation. Besides, immature large-scale production technology of CRISPR reagents, imperfect clinical trial design, as well as ethical and regulatory disputes over gene editing technology all hinder its clinical transformation. Notably, the limited stability of ribonucleoprotein (RNP) complexes, which can dissociate at elevated temperatures (Camperi et al., 2022), presents additional challenges for scalable production and storage.

To advance CRISPR technology for influenza prevention and control, critical bottlenecks must be addressed through targeted innovation. Delivery systems require major improvement. Optimizing the lipid composition of LNPs and developing novel ionizable lipids may substantially enhance endosomal escape and improve penetration across the respiratory mucosa (Kazemian et al., 2022). At the same time, biomimetic delivery carriers that integrate nanotechnology with cell-membrane cloaking strategies may improve targeting efficiency and biocompatibility by exploiting the natural properties of cell membranes (Wu et al., 2024). From a strategic perspective, it is important to develop integrated CRISPR platforms that combine Cas13 and Cas9. Cas13 directly cleaves viral RNA to suppress viral replication, while Cas9 edits host susceptibility genes to reduce cell susceptibility and enhance host antiviral defense capacity (Broksø et al., 2025). In addition, the incorporation of Cas12, Cas14, and related systems may broaden the target range, while multiplex crRNA design may help counter viral escape. For safety evaluation, a comprehensive assessment framework should combine long-term animal studies, organoid-based *in vitro* models (Xiaoshuai et al., 2022), and highly sensitive off-target detection methods to more accurately characterize long-term effects and off-target risks. To facilitate clinical translation, stronger collaboration among industry, academia, and research institutions will be needed, along with improved large-scale manufacturing processes for CRISPR components and reduced production costs. Equally important are well-designed clinical trials to define optimal dosing regimens, international cooperation for the sharing of data and expertise, more robust ethical and regulatory frameworks, and the gradual development of CRISPR-based technologies into a broadly effective, durable, and safe tool for influenza prevention and control.

## 5 Conclusion and prospects

CRISPR gene-editing technology, characterized by its programmability and precise targeting capabilities, has facilitated groundbreaking advances in the prevention and management of IAV and IBV. By addressing key limitations of traditional vaccines and pharmaceuticals, particularly in overcoming viral mutation and drug resistance, this technology has demonstrated substantial application value and broad translational potential (Wu et al., 2025). The technology operates through a dual mechanism of direct antiviral activity and host-directed defense. The RNA-targeting CRISPR-Cas13 system specifically cleaves highly conserved single-stranded RNA sequences within the genomes of IAV and IBV, while its collateral cleavage activity may further support broad-spectrum antiviral applications. Through technical optimization, the system can effectively mitigate potential cytotoxicity to host cells (Abudayyeh et al., 2016). In addition, the DNA-targeting CRISPR-Cas9 system reduces cellular susceptibility to viruses by knocking out host susceptibility genes, such as SLC35A1, thereby establishing a durable antiviral barrier (Han et al., 2018).

Despite persistent challenges related to delivery efficiency, long-term safety, viral escape, and clinical translation, ongoing technological innovation and multidisciplinary integration position CRISPR technology as a pivotal tool in influenza prevention and control. With continued refinement, this platform may offer broad-spectrum efficacy, durable activity, and improved safety, thereby contributing to global public health security.
